# Characterization of the complete chloroplast genome of *Hevea pauciflora* (Euphorbiaceae), an important wild relative of the rubber tree

**DOI:** 10.1080/23802359.2022.2115321

**Published:** 2022-09-07

**Authors:** Yan-Shi Hu, Hua-Sun Huang, Jin Liu

**Affiliations:** a Rubber Research Institute, Chinese Academy of Tropical Agriculture Science, Danzhou, China; b Yunnan Institute of Tropical Crops, Xishuangbanna, China

**Keywords:** *Hevea pauciflora*, chloroplast genome, characterization, phylogenetic analysis

## Abstract

*Hevea pauciflora* belongs to the Euphorbiaceae family, an important wild relative of the rubber tree. This study sequenced, assembled, and annotated the complete chloroplast genome of *H. pauciflora*. The complete chloroplast genome is 161,123 bp with a canonical quadripartite structure containing a large single-copy (LSC) region (89,109 bp), a small single-copy (SSC) region (18,376 bp), and two inverted repeat regions (IRa and IRb) (26,819 bp, each). A total of 134 genes were annotated, including 86 protein-coding genes, four pseudogenes, 36 tRNA genes, and eight rRNA genes. The 134 genes include four major groups: ‘self-replication’, ‘photosynthesis’, ‘unknown function’, and ‘others’. A phylogenetic analysis clustered *H. pauciflora*, *H. brasiliensis*, *H. camargoana*, and *H. benthamiana* into one clade, consistent with traditional taxonomy. This study provides useful data for further studies of *Hevea* genus and the phylogenetic relationships of Euphorbiaceae species.

*Hevea pauciflora* (Benth.) Mull. Arg., Linnaea 1865, belongs to the *Hevea* genus of the Euphorbiaceae family, an important wild relative of the rubber tree (*Hevea brasiliensis*). The rubber tree is the primary commercial source of high-quality natural rubber, an important industrial raw material. The rubber tree and *H. pauciflora* originated from the Amazon river basin of South America. Nonetheless, the current rubber tree planting area mainly covers Indonesia, Malaysia, Thailand, China, and other Southeast Asian countries. Genus *Hevea* consists of 11 species (Clément-Demange et al. 2007), including *H. pauciflora*, *H. brasiliensis*, *H. guianensis*, *H. camargoana*, *H. benthamiana*, *H. microphylla*, *H. rigidifolia*, *H. spruceana*, *H. paludosa*, *H. nitida*, and *H. camporum* (Goncalves et al. 1990; Priyadarshan and Goncalves 2002). All the 11 species are diploids with 36 chromosomes, except *H. pauciflora* and *H. guianensis*, with 18 and 54 chromosomes, respectively (Baldwin 1947; Majumder 1964).

The nuclear genome of *H. brasiliensis* has been completely assembled (Rahman et al. 2013; Lau et al. 2016; Tang et al. 2016; Pootakham et al. 2017; Liu et al. 2020). Meanwhile, the chloroplast genomes of *H. brasiliensis* (Tangphatsornruang et al. 2011), *H. benthamiana* (Niu et al. 2020), and *H. camargoana* (Niu et al. 2020) have been sequenced. *H. pauciflora*, the only species with 18 chromosomes in the genus *Hevea*, has great potential for distant hybridization breeding of the rubber tree. However, the chloroplast genome sequence of *H. pauciflora*, a crucial requirement for hybridization breeding, was lacking. Thus, this study sequenced and analyzed the chloroplast genome of *H. pauciflora*.

Healthy, young leaves of *H. pauciflora* (Figure 1) were collected from The Rubber Tree Germplasm Resource Nursery of the Chinese Academy of Tropical Agriculture Science (N 19°34′31.53″ and E 109°31′17.97″). High-quality genomic DNA was extracted from *H. pauciflora* leaves using the DNeasy Plant Mini Kit (Qiagen, Hilden, Germany), following the manufacturer’s instructions. The specimens and DNA samples were deposited in the herbarium and cryogenic sample library of the Yunnan Institute of Tropical Crops (http://www.yitc.com.cn, Dr. Jin Liu, liujin06@126.com), voucher numbers YITC-2020-FZ-E-115 and D2020-FZ-E-115, respectively.

**Figure 1.**
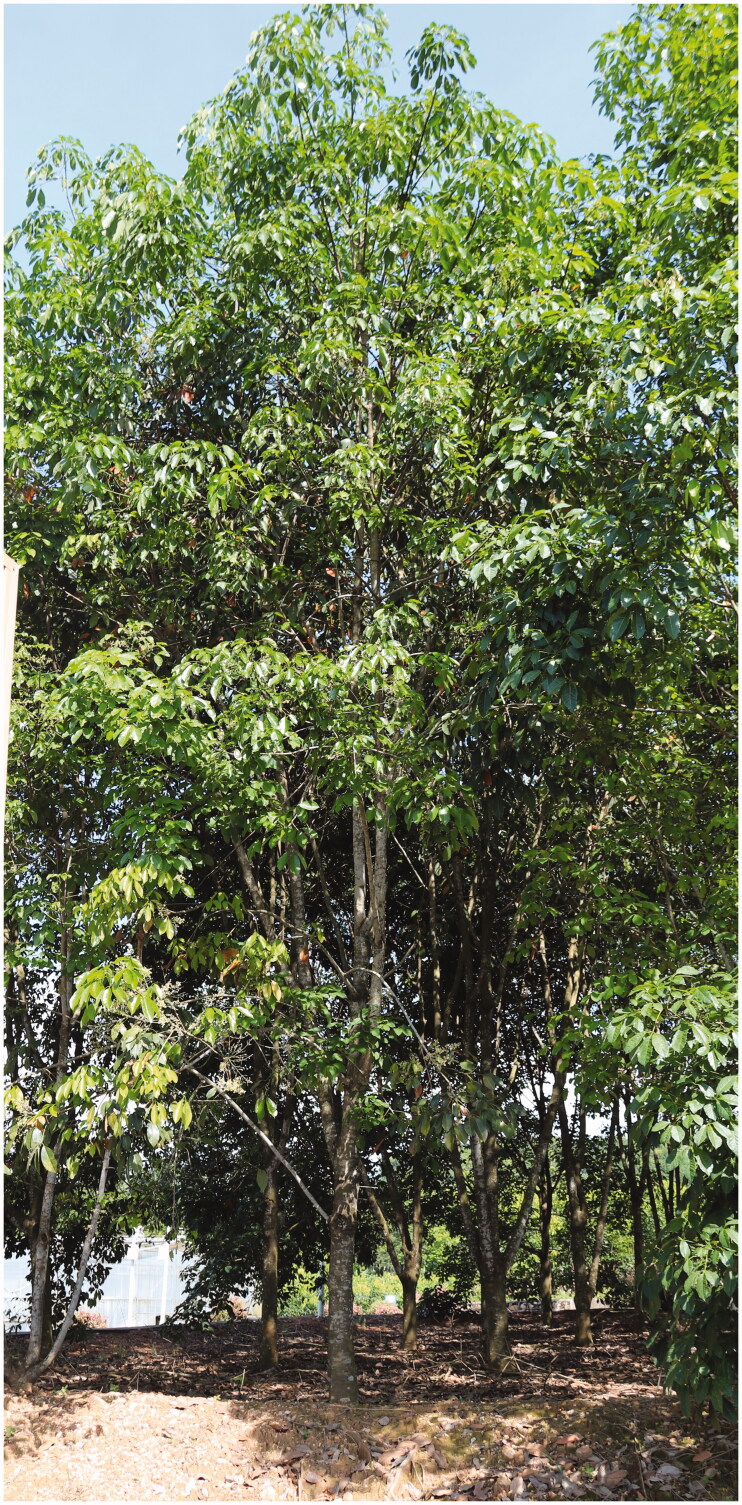
Species image of *H. pauciflora.*

The total DNA was used to produce paired-end (PE) Illumina sequencing libraries with 350 bp average insert size, and sequenced on the Illumina HiSeq 2500 platform (Illumina, San Diego, CA). The generated 7.5 Gb raw data were filtered and assembled using the SPAdes-3.5.0 (http://soap.genomics.org.cn/soapdenovo.html), following the sequence overlap and PE relationships. Sanger sequencing was applied to verify the four boundaries of the IR region and the chloroplast genome was annotated using CpGAVAS2 (Shi et al. 2019) and GeSeq (Tillich et al. 2017). The complete, annotated chloroplast genome was submitted to GenBank (http://www.ncbi.nlm.nih.gov/), accession number MW528030.

Next, the *H. pauciflora* chloroplast genome was phylogenetically analyzed using the maximum-likelihood method and 17 (Figure 2) other Euphorbiaceae species to understand the phylogenetic relationship between the 18 chloroplast genomes. The 17 Euphorbiaceae species included four genera: *Euphorbia* (10 species), *Hevea* (four species), *Croton* (two species), and *Deutzianthus* (one species). *Hydnocarpus hainanensis*, a tree species belonging to the Achariaceae family (order Malpighiales, as with the other 17 species), was the outgroup. Multiple sequence alignment was performed using MAFFT (Katoh and Standley 2013), whereas RAxML8.2.4 was employed to conduct phylogenetic analysis (Stamatakis 2014). Node support was estimated from the results of 1000 bootstrap replicates.

**Figure 2.**
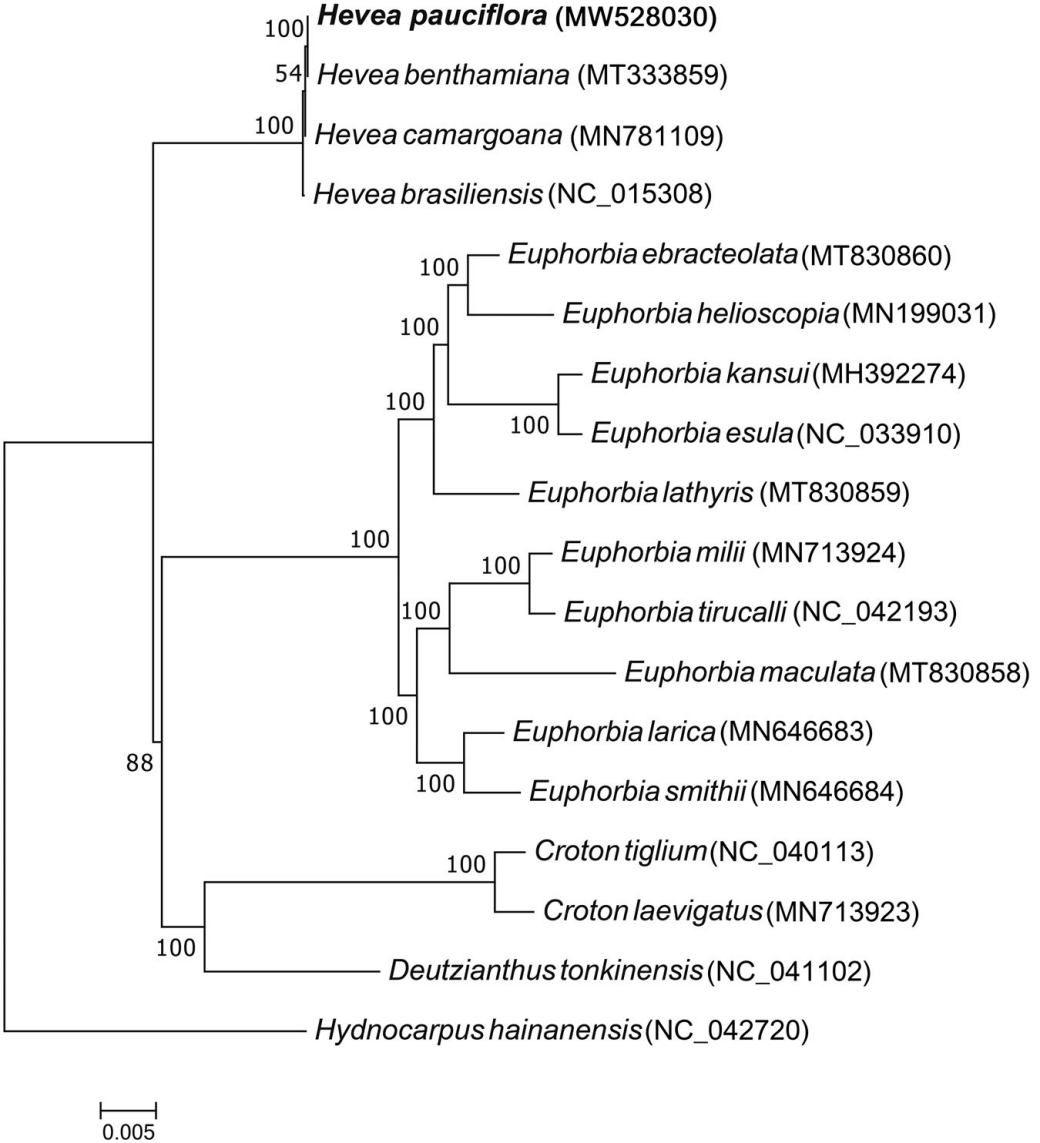
Maximum-likelihood tree based on complete chloroplast genome sequences of *H. pauciflora* and other Euphorbiaceae species. *Hydnocarpus hainanensis*, a tree species belonging to the Achariaceae family, was the outgroup.

The complete chloroplast genome of *H. pauciflora* is 161,123 bp long, with a canonical quadripartite structure (Figure 3). The structure contains a large single-copy (LSC) region of 89,109 bp (33.18% GC content), a small single-copy (SSC) region of 18,376 bp (29.42% GC content), and two inverted repeat regions (IRa and IRb) of 26,819 bp (42.20% GC content). Furthermore, the annotated *H. pauciflora* chloroplast genome contains 134 genes, including 86 protein-coding genes, 36 tRNA genes, eight rRNA genes, and four pseudogenes. The whole chloroplast genome contains 50,494 A bases (31.96%), 52,023 T bases (32.29%), 28,915 G bases (17.95%), and 28,691 C bases (17.81%). Moreover, the 134 genes contain four gene categories: self-replication, photosynthesis, unknown function, and other genes.

**Figure 3.**
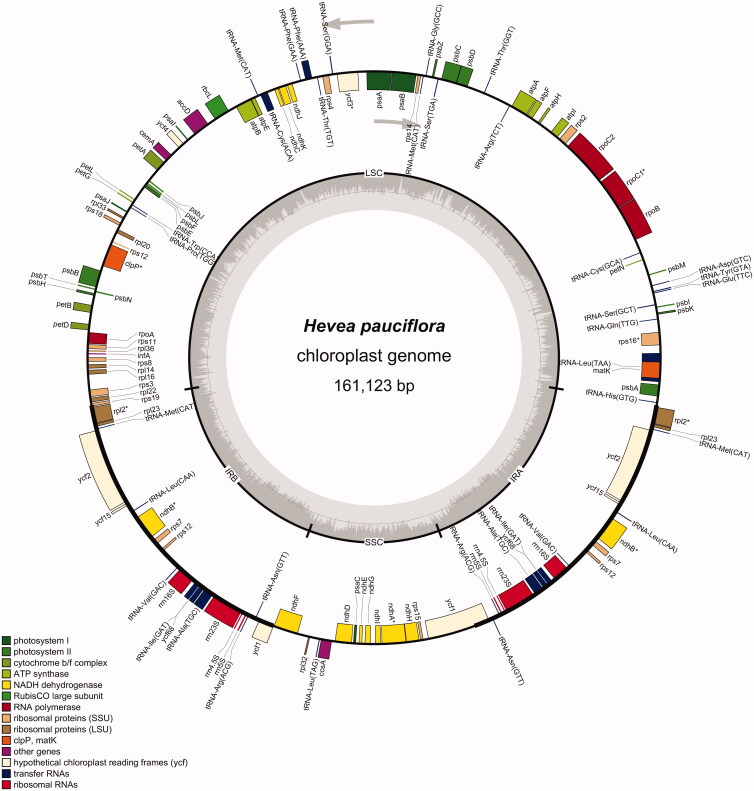
The chloroplast genome map of *H. pauciflora.*

Phylogenetic analysis showed that *H. pauciflora*, *H. brasiliensis*, *H. camargoana*, and *H. benthamiana* are closely clustered in one clade (Figure 2), consistent with traditional taxonomy. This *H. pauciflora* chloroplast genome sequence provides useful data for further studies of the *Hevea* genus and understanding the phylogenetic relationships of Euphorbiaceae species.

## Data Availability

The genome sequence data that support the findings of this study are openly available in GenBank of NCBI at https://www.ncbi.nlm.nih.gov/ under accession no. MW528030. The associated BioProject, SRA, and Bio-Sample numbers are PRJNA763296, SRR15911750, and SAMN21437637, respectively.
